# The WORKWELL programme: a randomized controlled trial of job retention vocational rehabilitation for people with inflammatory arthritis

**DOI:** 10.1093/rheumatology/keag373

**Published:** 2026-07-17

**Authors:** Alison Hammond, Jennifer Parker, Sarah Cotterill, Chris Sutton, Selman Mirza, Angela Ching, Terence W O’Neill, Fiona Holland, Antonia Marsden, Paula Holland, Kathryn Radford, Suzanne M M Verstappen, Martin Eden, Garima Dalal, Simone Battista, June Culley, Karen Walker-Bone, Sarah Woodbridge, Rachel O-Brien, Yvonne Hough, Yeliz Prior

**Affiliations:** Centre for Human Movement and Rehabilitation, School of Health and Society, University of Salford, Salford, UK; Arthritis UK/MRC Centre for Musculoskeletal Health and Work, University of Salford, Salford, UK; Centre for Human Movement and Rehabilitation, School of Health and Society, University of Salford, Salford, UK; Arthritis UK/MRC Centre for Musculoskeletal Health and Work, University of Salford, Salford, UK; Centre for Biostatistics, School of Health Sciences, The University of Manchester, Manchester, UK; Lancashire Clinical Trials Unit, Allied Health Research Hub, University of Central Lancashire, Preston, UK; Centre for Biostatistics, School of Health Sciences, The University of Manchester, Manchester, UK; Centre for Human Movement and Rehabilitation, School of Health and Society, University of Salford, Salford, UK; Arthritis UK/MRC Centre for Musculoskeletal Health and Work, University of Salford, Salford, UK; Centre for Epidemiology Versus Arthritis, Centre for Musculoskeletal Research, The University of Manchester, Manchester, UK; NIHR Manchester Biomedical Research Centre, Manchester University NHS Foundation Trust, Manchester Academic Health Science Centre, Manchester UK; Centre for Biostatistics, School of Health Sciences, The University of Manchester, Manchester, UK; Centre for Biostatistics, School of Health Sciences, The University of Manchester, Manchester, UK; Faculty of Health and Medicine, Lancaster University, Lancaster, UK; Centre for Rehabilitation and Ageing Research, School of Medicine, University of Nottingham, Nottingham, UK; NIHR Nottingham Biomedical Research Centre, Nottingham, UK; Centre for Epidemiology Versus Arthritis, Centre for Musculoskeletal Research, The University of Manchester, Manchester, UK; NIHR Manchester Biomedical Research Centre, Manchester University NHS Foundation Trust, Manchester Academic Health Science Centre, Manchester UK; Manchester Centre for Health Economics, The University of Manchester, Manchester, UK; Manchester Centre for Health Economics, The University of Manchester, Manchester, UK; Centre for Human Movement and Rehabilitation, School of Health and Society, University of Salford, Salford, UK; Arthritis UK/MRC Centre for Musculoskeletal Health and Work, University of Salford, Salford, UK; Patient Research Partner, Derby, UK; Monash Centre for Occupational and Environmental Health, Monash University, Melbourne, Australia; Arthritis UK/MRC Centre for Musculoskeletal Health and Work, University of Southampton, Southampton, UK; Centre for Human Movement and Rehabilitation, School of Health and Society, University of Salford, Salford, UK; Arthritis UK/MRC Centre for Musculoskeletal Health and Work, University of Salford, Salford, UK; School of Health and Social Care, Sheffield Hallam University, Sheffield, UK; Occupational Therapy, St Helens & Knowsley NHS Trust, St Helens Hospital, St Helens, UK; Centre for Human Movement and Rehabilitation, School of Health and Society, University of Salford, Salford, UK; Arthritis UK/MRC Centre for Musculoskeletal Health and Work, University of Salford, Salford, UK

**Keywords:** inflammatory arthritis, vocational rehabilitation, job retention, occupational therapy, randomized controlled trial, work productivity, presenteeism, employment outcomes, self-management, rheumatology rehabilitation

## Abstract

**Objective:**

People with inflammatory arthritis (InfA) frequently experience work instability, absenteeism, and reduced productivity, culminating in early job loss. This study evaluated the effectiveness and cost-effectiveness of WORKWELL job retention vocational rehabilitation (JRVR) delivered by occupational therapists, compared with a control group receiving written self-help advice.

**Methods:**

A pragmatic, multicentre randomized controlled trial was conducted across 18 UK National Health Service (NHS) Trusts. Employed adults (*n* = 249) with InfA experiencing moderate to severe work instability were randomized (1:1) to intervention or control groups. WORKWELL included structured work assessment, individually tailored action plans, and interventions over 2–4 months. Follow-up was at 6, 12 and 36 months. The primary outcome was measured using the Work Limitations Questionnaire-25 (WLQ-25). Analyses used linear mixed-effects regression adjusted for baseline characteristics and occupational skill level. A NHS perspective was used for the within-trial cost-effectiveness analysis. Much of the trial was affected by the COVID-19 pandemic.

**Results:**

At 12 months, there was no significant difference in WLQ-25 between groups (adjusted mean difference: −1.8; 95% CI −7.4–3.8; *P* = 0.53), or in most secondary outcomes at 12 or 36 months. However, absenteeism showed a relative reduction of 46% at 12 months (*P* = 0.08), and employment retention at 36 months was higher in the intervention (93%) than in the control group (85%). The intervention was not cost-effective from an NHS perspective.

**Conclusion:**

WORKWELL did not lead to improved work productivity compared with self-help advice. Future research should explore more flexible delivery methods, including digital tools, to support sustainable employment for people with InfA. A plain-language abstract is available in the supplementary material.

**Trial registration:**

ClinicalTrials.gov, http://clinicaltrials.gov, NCT03942783. ISRCTN Registry, ISRCTN61762297, https://doi.org/10.1186/ISRCTN61762297.

Rheumatology key messages

- WORKWELL wasn’t effective or cost-effective in the NHS, though wider societal benefits remain unmeasured.
- At 36 months, most participants in both groups remained in paid work, with no significant differences between the intervention and control arms.
- Future approaches to supporting work participation in people with inflammatory arthritis should explore more flexible, scalable delivery models, including digital self-help tools.

## Introduction

Work ability decreases in employed people with inflammatory arthritis (InfA). Among those with RA, 40% to 67% experience presenteeism (reduced work productivity), 30% to 84% absenteeism, and 50% stop working within 4.5 to 22 years of diagnosis, despite modern medication [1–3]. Employment rates have changed little from 1980 to 2022 [4].

Job retention vocational rehabilitation (JRVR) is a work-focused intervention helping employed individuals with health conditions stay in work by addressing job-related challenges through tailored support, workplace strategies, and self-management. As work impacts occur early, JRVR can potentially keep people with InfA working. EULAR recommends using the biopsychosocial framework of health to address work needs, sustain work ability and productivity, and prevent long-term absence [5]. Two recent systematic reviews highlighted inconsistent results of JRVR in InfA [6, 7]. Two Dutch trials of multidisciplinary JRVR (*n* = 150; 1–2 years follow-up) showed no evidence of effect on work outcomes [8–10]. In one, initially 40% of participants were on long-term sick leave, meaning JRVR was potentially too late [8]. In the other, initially 50% had little presenteeism and work instability (a mismatch between ability and job demands), suggesting JRVR was too early [10]. Two small UK trials (*n* ≤ 55) with short follow-up (6–9 months), recruiting participants with moderate to severe work instability indicated beneficial effects on presenteeism [11, 12]. Two longer US trials (2–4.5 years follow-up) showed reduced job loss [13, 14], but not reduced presenteeism [15]. Interventions in the latter four trials tested JRVR delivered by a single professional [either a vocational rehabilitation (VR) counsellor, occupational therapist or physiotherapist]. The findings suggest that tailored JRVR, delivered by a consistent professional with VR training, have some beneficial effects on work outcomes. Cost-effectiveness evidence is required for resource allocation decision-making [15].

Work participation is a complex interplay between biopsychosocial consequences of health-related, personal and environmental contextual factors [16]. These factors help explain the impact of arthritis on work participation and can modify the effects of work-focused interventions [17–19]. Factors influencing remaining in work and fewer work limitations include younger age (<50 years), higher education, shorter disease duration, no comorbidities, lower disease activity, less pain, less fatigue and better function, better psychological status, less physically and mentally demanding jobs, higher work self-efficacy, and workplace support [4, 20, 21]. EULAR guidance recommends including such factors in trials of work outcomes in InfA to examine their effects [22]. These findings highlight that work participation is determined by interacting clinical, personal and occupational factors, rather than disease status alone. Interventions aiming to improve work outcomes therefore need to address this broader context, including workplace demands, individual capacity, and self-management. Vocational rehabilitation interventions are designed to target these multiple determinants by supporting individuals to adapt work tasks, manage symptoms, and negotiate workplace adjustments.

The aims of this study were to: (i) examine the effects of WORKWELL JRVR on presenteeism and other work outcomes at 6, 12 and 36 months post-randomization among people with InfA experiencing work instability; (ii) investigate what contextual factors (if any) influenced the results; and (iii) assess the cost-effectiveness of WORKWELL for resource allocation decision-making.

## Method

### Study design

The WORKWELL trial was a definitive, pragmatic, multicentre superiority randomized controlled trial (RCT). Participants were recruited from 18 National Health Service (NHS) Trust rheumatology departments in England, Wales, and Scotland. The sites opened from February to August 2019, following each site’s approval. Initially, a 12-month follow-up was approved, with a 36-month follow-up approved later. The first participant was randomized in March 2019, and the last in February 2021. The last 12-month questionnaire was returned in March 2022, and the last 36-month questionnaire in March 2024. Trial and process evaluation protocols have been published [23, 24]. The CONsolidated Standards of Reporting Trials (CONSORT) reporting guidelines were followed [25, 26].

### Participants

Inclusion criteria were: aged ≥18 years, diagnosed with RA, early InfA (EInfA, i.e. not meeting full diagnostic criteria for RA but treated as if RA) or PsA by a rheumatology consultant; in paid work for ≥15 hours/week; and experiencing work instability [score ≥10 on the RA-Work Instability Scale (RA-WIS), i.e. at medium to high risk of work instability, meaning JRVR is appropriate] [27]. Exclusion criteria were on long-term sick leave (>4 weeks); retiring in ≤12 months; and already receiving JRVR. Rheumatology clinicians or therapists identified patients during appointments, from department databases, or medical or therapy records. Patients were provided with an invitation letter and information sheet. If interested, they were screened (in person or by telephone) by an NHS research practitioner, therapist, or member of the research team, and the study explained. Eligible patients provided written consent. The baseline questionnaire was completed at home and mailed to the trial manager. Freepost envelopes were provided throughout the trial for postal returns.

Recruitment primarily occurred through NHS rheumatology services. During the COVID-19 pandemic, when recruitment slowed due to reduced clinical capacity and there being fewer eligible patients in employment, additional participants were recruited through two national arthritis charities. These organizations disseminated study information to individuals with InfA who were invited to contact the research team directly for eligibility screening.

### Interventions

Both groups continued receiving usual care.

#### Control group

Participants received a work self-help information pack about coping at work, sources of help, and employment rights, with a letter encouraging them to read the booklets enclosed, identify work problems and find solutions [24].

#### Intervention group

Participants in the intervention group received the same information pack. WORKWELL sessions were held in person, with flexible appointment times, usually at the applicable therapy department. JRVR included a structured work assessment [the Work Experience Survey–Rheumatic Conditions (UK WES-RC)] [28], prioritized goals, and tailored support, including self-management, job accommodations, and employer communication. Up to four sessions were delivered over 2–4 months, followed by a brief telephone review. A work site visit and/or letters for employers about work support recommended were offered, if required [23, 24].

### Therapist training

Occupational therapists, with experience supporting employed people with InfA, delivered WORKWELL. Beforehand, therapists completed a JRVR training programme, and demonstrated competency in a mock WES-RC and JRVR plan. Ongoing support was available via mentoring, peer discussion by email, and the WORKWELL Solutions Manual (including biopsychosocial strategies for addressing work-related problems) [29].

### Randomization, procedures and blinding

Following baseline questionnaire receipt, participants were assigned 1:1 to intervention or control groups, and stratified by job skill level (i.e. low: categories 1 to 2; or high: categories 3–4) using the Office for National Statistics classification [30]. Job skill level was selected as a stratification factor, because occupational demands and job control are known to influence work functioning and the ability to remain in employment among people with InfA. Stratification was by permuted blocks of random sizes (4, 6 or 8) conducted by the Lancashire Clinical Trials Unit (LCTU) using Sealed Envelope, a web-based central randomization service [31]. Participants were allocated to both groups from the start of recruitment at each site. After randomization, the LCTU mailed all participants the information pack and referred WORKWELL participants to the relevant therapist. Therapists contacted participants within 5 days of referral, and JRVR began within 4 weeks. Participating therapists were unblinded to group allocation. Data management staff were blinded to group allocation, as was the trial statistician when conducting the primary outcome analysis at 12 months [23].

### Data collection

The postal baseline questionnaire collected demographic (e.g. age, sex, education), employment (e.g. job title, hours worked, organization size) and health and health-care resource use information. This included detailed information on prescribed medications (conventional synthetic DMARDs, biologic and targeted therapies, and CSs). All outcomes were collected using validated self-reported questionnaires widely used in occupational health and rheumatology research. These instruments are designed for self-completion and capture perceived work limitations, work instability, and productivity. Data quality was supported through structured follow-up procedures, including reminders and telephone completion of minimum datasets where questionnaires were not returned.

Outcomes were collected using questionnaires at 6 months, 12 months, and 36 months. Follow-up questionnaires were available as mailed paper copies or online, with an e-mailed secure weblink. If not returned, after 2 weeks, participants were sent a text/email/telephone reminder, and at 3 weeks a second questionnaire copy or weblink. At 4 weeks, if not returned, trial managers attempted to collect a minimum dataset by telephone [23]. Intervention participants not completing JRVR continued in follow-up unless they withdrew.

### Outcomes

The primary outcome, at 12 months post-randomization, was the Work Limitations Questionnaire-25 (WLQ-25PDmod), a measure of presenteeism [32], as modified in 2016 [33], using the summed score of the four subscales. Secondary outcomes included these WLQ sub-scales, WLQ-25PDMod productivity loss score, and the Workplace Activities Limitations Scale (WALS-12, 12 items, measuring work ability) [21]. The WLQ-25PDmod and WALS-12 were collected using the dual-key scored outcome measure. Both were scored using the original and dual-key methods, as the latter is more sensitive to change [34].

The scales used in this study have established scoring ranges and directions of interpretation. For the WLQ-25PDmod, higher scores indicate greater work limitations and reduced productivity. The WALS-12 ranges from 0 to 36, with higher scores reflecting greater difficulty performing work activities. The RA-Work Instability Scale (RA-WIS) ranges from 0 to 23, with higher scores indicating greater risk of work instability. The Work Self-Efficacy (WSE) scale measures confidence in managing work demands, with higher scores indicating greater self-efficacy. The RA Impact of Disease (RAID) and RA-Disease Activity Index-5 (RADAI-5) both range from 0 to 10, with higher scores indicating greater disease impact or activity. For all measures, negative change scores represent improvement whereas higher scores denote worse outcomes.

Other secondary outcomes were the Work Productivity and Activity Impairment questionnaire v2.0 (WPAI) (0 months, 6 months, 12 months) [35]; employment status; absenteeism (number of days off sick, collected monthly by text, email or telephone) (0 through 12 months); the RA-Work Instability Scale (RA-WIS) (0 months, 12 months) [27]; WSE (0 months, 12 months); and satisfaction with work support received (6 months).

Analysis of contextual factors, potentially influencing the intervention’s impact on the primary outcome, included (0 months, 12 months): the Short-Form12v2 Health Survey (SF12v2) [36], RAID [37] and RADAI-5 [38]; job skill level (0 months, 12 months, 36 months) [30]; Work Demands (WD) (0 months, 12 months) [39]; and Rheumatic Disease Comorbidities Index (0 months) [40]. Although RA-specific instruments were originally developed for RA populations, they capture domains such as pain, fatigue, function, and disease impact that are common across InfA conditions, including early InfA and PsA. These instruments have therefore been used in mixed InfA cohorts to provide consistent patient-reported outcome measurement.

For a within-trial cost-effectiveness analysis (CEA), the EQ-5D-5L questionnaire was used (0 months, 6 months, 12 months) to determine participants’ health-related quality-of-life (HRQoL) [41], and NHS resource use was determined via a participant questionnaire. This included use of hospital services (e.g. inpatient/outpatient/day case/A&E), use of primary/community-based services (e.g. General Practice/mental health services/occupational therapy), and any prescribed medication. Resource use associated with delivering the intervention was determined from trial documentation completed by therapists.

### COVID-19 effects on trial and impacts

The trial paused in March 2020 for safety reasons, with JRVR suspended. To enable re-start, trial adaptations were discussed with the Patient and Public Involvement Group (PPIG) and therapists. Following site approval, 13 sites re-started in July or August 2020, and 5 between September 2020 and January 2021. Periodic lockdowns (restrictions on the public’s movements) continued until April 2021. Non-essential workers worked remotely from home (WRFH) until January 2022. If unable to WRFH, they could be furloughed (the Coronavirus Job Retention Scheme) until September 2021. Essential workers continued attending work throughout. Participants in follow-up received questionnaires to schedule. At 6 months and 12 months, most participants completed questionnaires while COVID-19 restrictions were in place [6 months: *n* = 174 (80%); 12 months *n* = 192 (94%)].

Following re-start, recruitment slowed due to reduced site staff availability and fewer eligible patients (due to job loss or furlough). Additional participants were recruited via two arthritis charities to reach the target sample size.

At restart, the trial website included downloadable documents for electronic completion, and an online Workwell Solutions Manual. JRVR was delivered remotely by telephone or videoconferencing. In-person treatment became possible later. Two therapists in the research team provided participants’ JRVR remotely for seven sites, due to therapist redeployment or sickness, and treated charity-recruited participants. Normally, participants were treated by one therapist, or occasionally by a second if the original was unavailable [42].

### Sample size

To identify a minimal clinically important difference (13 points) [43] in the primary outcome at 12 months, assuming 90% power, s.d. = 23.02 (conservative estimate from our feasibility trial [12, 23]), 5% significance level, and an intra-class correlation coefficient (ICC) of 0.05 in the intervention arm to account for a potential therapist clustering effect [with an average of 5 (variance = 5) participants per therapist], 90 participants were needed in each group [24]. Allowing for a 25% drop-out, the recruitment target was 240.

### Statistical analysis

The analysis followed a pre-specified Statistical Analysis Plan (SAP) and was by intention-to-treat, using STATA version 17 [44]. (There was a minor deviation from the SAP. We originally analysed the 6- and 12-month results in a two time point longitudinal model, in a blinded analysis, as described in the SAP, and we planned to analyse the 36-month outcomes in a separate one time point model. However, we later decided to publish all time points together in this article, and to avoid confusion, we analysed the 6-,12- and 36-month outcomes in one three time point model, which could not be blinded. The findings were not sensitive to the model fitted, so this has not led to any changes in the results at any time point.)

Baseline characteristics were described overall and by group, reporting mean (s.d.), median [interquartile range (IQR)] or number (proportion) as appropriate. Primary effectiveness analysis used a linear mixed-effects (lme) regression model to estimate the effect of group allocation on WLQ-25PDmod scores at 6 months, 12 months and 36 months, controlling for the stratification variable (job skill level) and baseline value of the WLQ-25PDmod. A participant-specific random effect nested within a therapist-level random effect was specified. As the therapist effect only occurred in those who received the WORKWELL intervention, this was a partially nested design. Therapist effect was allocated by the therapist seen most frequently. The model estimated the variance of residual errors separately in the intervention groups to allow for between-group heteroscedasticity. Outcomes were analysed in a single model, with an interaction term between time point and intervention group to determine the treatment effect at each time point separately. Of primary interest was the treatment effect at 12 months. Sensitivity analyses using alternative definitions of per-protocol analyses were conducted: (1) excluding intervention participants not receiving JRVR, and control participants reporting at 12 months receiving JRVR; and (2) excluding intervention participants not receiving JRVR or only attending the first treatment appointment (i.e. work assessment), and control participants reporting receiving JRVR. Secondary outcome analyses also used appropriate generalized lme modelling methods (i.e. linear, logistic, or ordinal logistic), controlling for the stratification variable, baseline value of that outcome variable, and a random therapist effect. The interaction between time point and group was not included for measures collected post-randomization at 12 months only.

We considered the association of various contextual factors with the primary outcome in an exploratory analysis, adding a three-way interaction term (time point, intervention group, contextual factor) into the main model.

The primary within-trial economic evaluation followed the NICE reference case [45]. Published unit costs were attached to reported NHS resource use. Quality-adjusted life-years (QALYs) were calculated using utility scores mapped to reported EQ-5D-5L states [46]. Missing cost and outcomes data were imputed using multiple imputation by chained equations in STATA. Costs and QALYs were adjusted for previous NHS resource use prior to trial enrolment and baseline HRQoL, respectively. An incremental cost-effectiveness ratio (ICER) was calculated using the following:


$$\text{ICER}\;=\;\frac{(\text{Cost}\;\text{of}\;\text{JRVR}—\text{Cost}\;\text{of}\;\text{Control})\;}{\left(\text{Effectiveness}\;\text{of}\;\text{JRVR}—\text{Effectiveness}\;\text{of}\;\text{Control}\right)}$$

The ICER represents the cost of generating an additional QALY using JRVR. An ICER under the willingness-to-pay threshold of £20–30 000 is deemed cost-effective under current decision rules [45]. Using the lower £20 000 bound of threshold, an incremental net monetary benefit (INMB) was determined using the following:


$$\text{INMB}\;=\;\frac{£20k\times \text{Difference}\;\text{in}\;\text{Effectiveness}\;\text{between}\;\text{JRVR}\;\text{and}\;\text{Control}}{\text{Difference}\;\text{in}\;\text{Cost}}$$

A positive INMB indicates that JRVR is cost-effective from an NHS and Social Care perspective. To generate CIs around the ICER and INMB point estimates, bootstrapping was used to recalculate costs and outcomes in 1000 resamples [47].

## Results

### Recruitment and response rates

**Figure 1:**
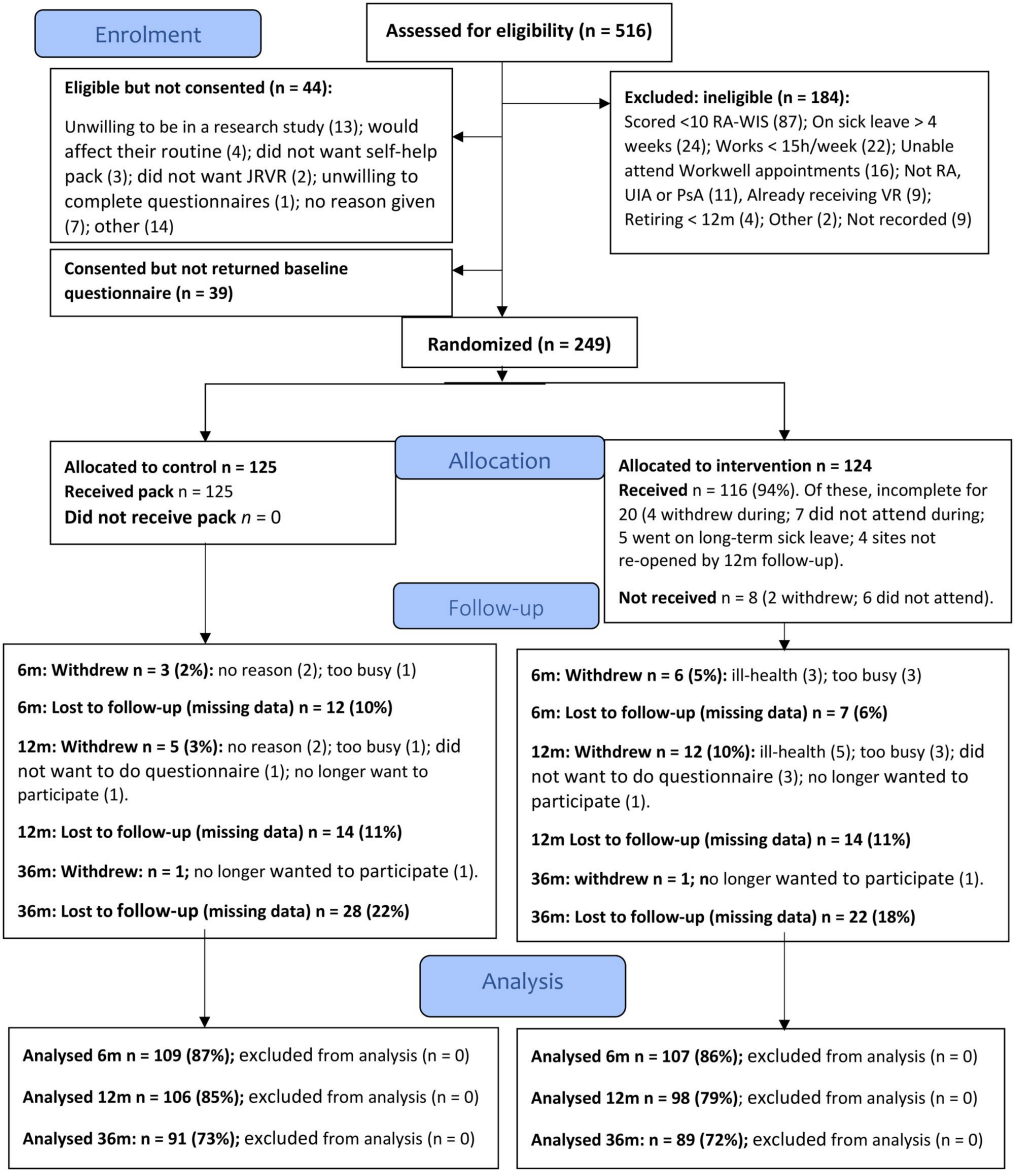
Workwell CONSORT Flow Diagram. JRVR: job retention vocational rehabilitation; RA-WIS: RA-Work Instability Scale

Fig. 1 shows participant flow through the trial: 249 were randomized (control = 125; intervention = 124). Of these, 34 (14%) were recruited via charities. All participants were mailed the self-help information pack. Questionnaire response rates were: 216 (87%) at 6 months; 204 (82%) at 12 months; and 180 (72%) at 36 months.

### Participants

The participant mean age was 49 years, 81% were women, median time since diagnosis was 4.4 years, and 58% worked full-time. The demographic and employment characteristics are in Table 1. At baseline, the mean WLQ-25PDmod summed score (0–100) was 30.9 (s.d. 17.0) in the control, and 30.8 (s.d. 19.8) in the intervention groups, i.e. low to moderate presenteeism (Table 2). Baseline disease characteristics are presented in Supplementary Table S3. Participants had moderate disease impact at study entry, with mean RAID scores of ∼6 and RADAI-5 scores of between 5 and 6, indicating ongoing disease burden despite treatment. Approximately one-third of participants had early disease (≤2 years duration). Participants had a mean duration of current employment of 11.0 years (s.d. 9.8), suggesting relatively stable employment at study entry. The majority of participants were employed on permanent contracts (95.2%), indicating a relatively stable employed population at study entry.

**Table 1:** Baseline participant demographic and employment characteristics (*n* = 249).

	Control (*n* = 125)	Intervention (*n* = 124)	Overall (*n* = 249)
**Demographic:**			
Age (years), mean (s.d.)	48.3 (10.2)	48.8 (9.5)	48.6 (9.9)
Sex (female/male/prefer not to say), *n* (%)	105 (84.0)/19 (15.2)/1 (0.8)	97 (78.2)/26 (21.0)/1 (0.8)	202 (81.1)/45 (18.1)/2 (0.8)
Diagnosis, *n* (%)			
-RA	81 (64.8)	84 (67.7)	165 (66.3)
-Early InfA	10 (8.0)	13 (10.5)	23 (9.2)
Time since diagnosis (years), median (IQR)	4.0 (1.4, 10.5)	5.0 (1.2, 11.3)	4.4 (1.3, 10.6)
Symptom duration (years), median (IQR)	6.0 (2.8, 13.0)	6.5 (2.8, 13.3)	6.0 (2.8, 13.3)
Medication regimen, *n* (%)			
-0 DMARD/biologic/biosimilar	13 (10.4)	6 (4.8)	19 (7.6)
-1 DMARD	37 (29.6)	45 (36.3)	82 (32.9)
-2 or more DMARDs	30 (24.0)	27 (21.8)	57 (22.9)
-Biologics/biosimilars (+/−DMARD)	45 (36.0)	46 (37.1)	91 (36.6)
Living status, *n* (%)			
-Living alone	22 (17.6)	21 (16.9)	43 (17.3)
-Living with family/spouse/significant other(s)	103 (82.4)	103 (83.1)	206 (82.7)
Education level, *n* (%)			
-Low education (ISCED 0–2)	34 (27.2)	24 (19.4)	58 (23.3)
-Medium education (ISCED 3–4)	28 (22.4)	23 (18.6)	51 (20.5)
-High education (ISCED 5–8)	62 (49.6)	76 (61.3)	138 (55.4)
-Missing	1 (0.8)	1 (0.8)	2 (0.8)
Ethnic group, *n* (%)			
-White	122 (97.6)	120 (96.8)	242 (97.2)
-Mixed/multiple ethnic	1 (0.8)	1 (0.8)	2 (0.8)
-Asian/Asian British	1 (0.8)	1 (0.8)	2 (0.8)
-Black/African/Caribbean/Black British	0 (0.0)	0 (0.0)	0 (0.0)
-Other	1 (0.8)	1 (0.8)	2 (0.8)
-Prefer not to say	0 (0.0)	1 (0.8)	1 (0.4)
**Employment:**			
Employment status, *n* (%)			
-Full-time (≥35 hours/week)	69 (55.2)	75 (60.5)	144 (57.8)
-Part-time (<35 hours/week)	56 (44.8)	49 (39.5)	105 (42.2)
No. of hours contracted to work/week, mean (s.d.)	30.9 (8.6)	33.2 (8.3)	32.1 (8.5)
No. of hours normally work/week, mean (s.d.)	33.2 (9.0)	35.2 (10.1)	34.2 (10.1)
Job contract/type, *n* (%)			
-Permanent contract	108 (86.4)	111 (89.5)	219 (88.0)
-Fixed-term contract	4 (3.2)	3 (2.4)	7 (2.8)
-Zero hours contract	1 (0.8)	0 (0.0)	1 (0.4)
-Agency contract	1 (0.8)	2 (1.6)	3 (1.2)
Self-employed	11 (8.8)	8 (6.5)	19 (7.6)
ONS Job Skill level, *n* (%)			
-1 (lowest)	9 (7.2)	7 (5.6)	16 (6.4)
-2	49 (39.2)	51 (41.1)	100 (40.2)
-3	24 (19.2)	32 (25.8)	56 (22.5)
-4 (highest)	43 (34.4)	34 (27.4)	77 (30.9)
Sole income earner (yes), *n* (%)	40 (32.0)	46 (37.1)	86 (34.5)
Organization size (people), *n* (%)			
-1 person (self)	9 (7.2)	6 (4.8)	15 (6.0)
-Micro (2–9)	9 (7.2)	6 (4.8)	15 (6.0)
-Small (10–49)	12 (9.6)	16 (12.9)	28 (11.2)
-Medium (50–249)	21 (16.8)	17 (13.7)	38 (15.3)
-Large (≥250)	74 (59.2)	79 (63.7)	153 (61.5)
Belong to a Trade Union/professional organization (yes), *n* (%)	52 (41.6)	51 (41.1)	103 (41.4)
Disclosed condition to employer (yes/no), *n* (%)	114 (91.2)/2 (1.6)	113 (91.1)/4 (3.2)	227 (91.1)/6 (2.4)
-Not applicable *n*, (%)	9 (7.2)	7 (5.6)	16 (6.4)
Occupational Health available in main job (yes), *n* (%)	68 (57.1)	71 (58.7)	139 (57.9)

+ Medication regimen reflects the number of DMARDs and/or biologic/biosimilar agents. Detailed medication classes (including conventional synthetic DMARDs, targeted therapies, biologics and CSs) were collected at baseline but are not presented here. InfA: inflammatory arthritis; IQR: interquartile range; ISCED: International Standard Classification of Education [ISCED 0—2 = no formal qualifications through to lower secondary education, e.g. General Certificate of Secondary Education (GCSE); 3–4 = upper secondary, and post-secondary non-tertiary education, e.g. A Levels, Business and Technology Education Council (BTEC), City & Guilds; 5–8 = short-cycle tertiary (e.g. diploma), Bachelor/Master/PhD degrees]; ONS: Office for National Statistics, Skill level: 1 = elementary occupations, 2 = administrative, caring, leisure, sales, customer service, process, plant, and machine operatives, 3 = skilled trades, associated technical and professional, 4 = professional, and managerial.

**Table 2:** Work outcome measures.

Outcome, mean (s.d.) unless otherwise stated:	Time (months)	*n*	Control	*n*	Intervention	Adjusted difference in means (95% CI)	*P*
WLQ-25PDmod Summed score (0–100)	0	125	30.9 (17.0)	124	30.8 (19.8)		
	6	101	32.2 (21.9)	88	27.9 (18.1)	−4.6 (−10.2, 1.1)	0.11
	12	98	32.4 (22.6)	91	30.4 (21.5)	−1.8 (−7.4, 3.8)	0.53
	36	70	35.2 (24.6)	70	31.6 (21.8)	−3.0 (−9.4, 3.5)	0.37
WLQ-25 Productivity Loss (%)	0	121	8.2 (4.4)	122	8.0 (5.1)		
	6	96	8.7 (5.8)	86	7.3 (4.9)	−1.3 (−2.8, 0.3)	0.11
	12	95	8.5 (5.9)	89	8.1 (5.7)	−0.6 (−2.1, 0.9)	0.45
	36	67	9.3 (6.2)	69	8.2 (5.6)	−1.0 (−2.8, 0.8)	0.26
WLQ subscales (0–100):							
-Time demands	0	124	35.1 (20.5)	124	35.0 (23.2)		
	6	100	31.0 (21.6)	88	28.6 (20.1)	−2.6 (−8.8, 3.7)	0.42
	12	98	35.5 (25.8)	91	32.1 (24.1)	−2.7 (−9.0, 3.5)	0.39
	36	70	39.0 (28.1)	70	32.6 (25.5)	−5.6 (−12.8, 1.5)	0.12
-Physical demands (modified)	0	122	39.6 (22.1)	123	39.6 (24.1)		
	6	98	40.8 (23.9)	86	36.5 (20.9)	−3.6 (−9.4, 2.2)	0.22
	12	95	41.2 (24.4)	89	36.2 (21.9)	−4.5 (−10.3, 1.2)	0.12
	36	69	42.9 (26.5)	69	40.3 (27.2)	−0.4 (−7.0, 6.2)	0.91
-Mental-interpersonal demands	0	125	24.2 (17.7)	124	24.0 (20.3)		
	6	101	28.9 (26.1)	88	23.2 (21.8)	−6.1 (−12.7, 0.4)	0.07
	12	98	27.1 (25.0)	91	26.8 (23.7)	−0.6 (−7.1. 6.0)	0.86
	36	70	29.0 (25.6)	70	27.2 (22.4)	−1.9 (−9.5, 5.7)	0.63
-Output demands	0	124	30.1 (20.1)	123	29.1 (22.1)		
	6	99	32.3 (27.1)	88	26.2 (23.1)	−5.9 (−13.0, 1.2)	0.10
	12	98	30.8 (26.9)	91	29.4 (25.1)	−1.7 (−8.7, 5.4)	0.64
	36	68	34.8 (26.9)	70	29.6 (22.2)	−5.0 (−13.2, 3.2)	0.23
Dual WLQ-25PDmod (0–100)	0	121	29.7 (15.4)	120	28.7 (18.0)		
	6	96	27.3 (16.6)	85	24.5 (13.5)	−2.0 (−6.0. 1.9)	0.31
	12	95	27.3 (17.6)	86	26.3 (16.5)	−1.5 (−5.4, 2.5)	0.46
	36	65	32.1 (20.9)	68	28.9 (18.7)	−2.7 (−7.1, 1.8)	0.25
WALS-12 (0–36)	0	125	12.2 (5.4)	124	12.0 (5.3)		
	6	101	10.8 (5.7)	88	10.6 (5.3)	−0.0 (−1.4, 1.3)	0.99
	12	98	11.7 (6.0)	91	10.7 (5.9)	−0.9 (−2.2, 0.5)	0.22
	36	72	13.9 (7.3)	74	11.9 (6.1)	−1.4 (−3.0, 0.1)	0.07
Dual WALS-12 (0–100)	0	109	35.7 (17.2)	111	34.4 (18.0)		
	6	84	31.3 (19.2)	77	30.2 (17.1)	−1.4 (−6.1, 3.3)	0.56
	12	85	33.7 (19.9)	77	31.8 (17.9)	−1.4 (−6.0, 3.3)	0.56
	36	59	38.4 (21.9)	64	35.6 (20.8)	0.1 (−5.1, 5.3)	0.97
Numbers in employment, *n*^a^							
Full-, part-time, unemployed	0	125	69/56/0	124	75/49/0		
	6	109	61/48/0^b^	107	60/46/1^c^		
	12	106	56/47/3^d^	98	59/37/2^e^		
	36	91	44/33/14^f^	89	49/34/6^g^		
**Data collected at 0, 6 and 12 months only**							
WPAI, past 7 days:							
-% work time missed due to health problems	0	124	5.4 (10.9)	116	6.1 (13.8)		
	6	100	3.4 (10.7)	83	2.9 (10.7)	−0.7 (−3.6, 2.1)	0.61
	12	95	2.4 (7.5)	87	3.4 (9.6)	1.0 (−1.9, 3.8)	0.50
-% impairment while working due to health	0	124	45.9 (22.8)	118	45.3 (23.5)		
	6	99	40.4 (24.9)	88	43.1 (23.9)	2.5 (−3.8, 8.7)	0.44
	12	96	42.0 (25.1)	87	38.9 (23.2)	−3.9 (−10.2, 2.4)	0.23
-% overall work impairment due to all health	0	124	47.8 (23.7)	116	47.6 (24.6)		
	6	99	42.0 (25.3)	83	43.3 (25.3)	1.4 (−5.2, 7.9)	0.68
	12	95	43.2 (25.8)	87	40.4 (24.4)	−4.4 (−10.9, 2.2)	0.19
-% activity impairment due to all health	0	125	62.8 (22.8)	124	58.1 (25.0)		
	6	109	49.5 (27.8)	106	51.1 (25.3)	4.3 (−2.3, 10.9)	0.20
	12	104	48.8 (26.2)	97	46.2 (25.1)	0.4 (−6.4, 7.2)	0.91
**0 and 12 months only**							
Absenteeism							
-Participant monthly self-report of number of days off-sick	6	121	10. (23.6)	118	7.5 (17.0)	−30% (−64%, 37%)^h^	0.30
	12	111	9.5 (16.9)	109	8.5 (24.3)	−46% (−73%, 7%)^h^	0.08
RA-WIS (0–23)	0	125	16.2 (4.2)	124	15.8 (4.6)		
	12	97	14.9 (5.5)	90	14.4 (5.5)	−0.3 (−1.5, 0.9)	0.64
Work Self-Efficacy (0–30)	0	125	17.9 (5.3)	124	18.0 (5.5)		
	12	102	18.4 (6.7)	93	19.1 (6.)	0.4 (−1.0, 1.8)	0.56
**Exploratory outcomes:**							
Satisfaction with work advice received in trial (0–10)	6	105	7.2 (2.5)	102	8.7 (1.6)	–	0.001

a Employment status: of those in employment, numbers not attending work due to shielding/furlough/sick leave/authorized leave; at 6 months:

b Control = 6,

c Intervention = 10;

d at 12 months: Control = 6,

e Intervention = 6; at 36 months,

f Control = 7;

g Intervention = 5;

h % relative reduction in sick leave of intervention group compared with control group.

RA-WIS; Rheumatoid Arthritis—Work Instability Scale; WALS: Work Activity Limitations Scale; WLQ-25PDmod: Work Limitations Questionnaire-25: Modified Physical Demands scale (i.e. items are not reverse scored)); WPAI Work Productivity Activity Impairment.

### Intervention provision

Overall, 96/124 (77%) completed JRVR, of whom: 30/33 were treated before the pandemic, 24/36 were paused participants, and 42/55 were recruited after re-start (Supplementary Table S1). Of 355 appointments, 189 (53%) were in-person, 106 (30%) telephone, and 60 (17%) videocall. Twenth-six (21%) participants were treated by research team therapists. On average, participants received 3.6 (s.d. 1.5) appointments. The work barriers identified are detailed elsewhere [31].

Intervention delivery was not contingent on continued employment, and participants were able to continue receiving the intervention where appropriate. During the trial, a small proportion of participants reported changes in employment status; however, most remained in work at 12 months (97.2% control; 98.0% intervention).

### Primary outcome

The regression model was analysed longitudinally based on 219/249 (88%; 114 control and 105 intervention) of the randomized participants with a baseline and at least one 6-month, 12-month or 36-month follow-up score. At 12 months, the primary end point, after adjusting for the stratification variable and baseline scores, there was no evidence of an effect of the intervention on presenteeism, compared with the control group [adjusted mean difference of −1.8 (95% CI: −7.4 to 3.8), *P* = 0.53]. While the point estimate of −1.8 favoured the intervention group, the lower confidence limit (−7.4) did not extend beyond the clinically important difference of −13. At 36 months, the trial also found no evidence of an effect (−3.0, 95% CI −9.4 to 3.5, *P* = 0.37). The results are shown in Table 2 and the sensitivity analyses are shown in Supplementary Table S2.

### Secondary outcomes

Across the secondary outcomes, at all three time points, the mean differences between the two groups were mostly small and, while favouring the intervention group, were not statistically significant. Those approaching statistical significance were the WLQ-25 mental-interpersonal demands scale (6 months) (*P* = 0.07), absenteeism, with a relative reduction of 46% (12 months) (95% CI: 73% reduction to a 7% relative increase, *P* = 0.08), and the WALS-12 (36 months) (*P* = 0.07) (Table 2). Health outcomes remained stable from 0 to 12 months (Table 3). At 12 months, satisfaction with work advice received was higher in the intervention group (8.7/10, s.d. 1.6) than in the control group (7.2/10, s.d. 2.5). At 12 months, most participants remained employed, although by 36 months employment was higher in the intervention group (93%) compared with the control group (85%). The proportion changing jobs due to health was higher at 36 months in the intervention group (15%) than in the control group (7%).

**Table 3:** Health secondary outcome measures.

Outcome, mean (s.d.)	Time (months)	*n*	Control	*n*	Intervention	Adjusted difference in means (95% CI)	*P*
SF-12v2: Physical Component Score (0–100)	0	125	36. (7.5)	124	36.9 (7.9)		
	6	107	37.0 (8.8)	102	37.8 (8.8)	0.1 (−1.8,1.9)	0.94
	12	101	39.7 (9.5)	89	39.6 (8.8)	−1.1 (−3.0, 0.8)	0.24
SF-12v2: Mental Component Score (0–100)	0	125	41.0 (9.1)	124	41.6 (9.7)		
	6	107	44.6 (9.3)	102	44.1 (9.4)	−1.0 (−3.3, 1.2)	0.37
	12	101	41.5 (10.1)	89	42.3 (10.1)	0.1 (−2.3, 2.4)	0.95
RAID (0–10)	0	125	6.2 (1.7)	124	5.8 (1.8)		
	12	101	5.6 (2.2)	89	5.3 (1.9)	0.1 (−0.4, 0.5)	0.76
RADAI-5 (0–10)	0	125	6.1 (1.8)	124	5.6 (1.8)		
	12	101	5.3 (2.1)	89	5.2 (1.8)	0.0 (−0.5, 0.5)	0.87

RAID: Rheumatoid Arthritis Impact of Disease; RADAI-5: Rheumatoid Arthritis Disease Activity Index-5; SF-12v2: Short Form 12v2 Health Survey.

### Contextual factors analysis

Most personal, environmental and health-related variables explored were not found to be contextual factors influencing the level of the primary outcome. There was a trend towards a significant interaction for Work Demands—Help from Colleagues at 36 months (*P* = 0.08), and a significant interaction for Work Demands—Mentally Demanding Work (*P* = 0.02). However, the group with little/no mentally demanding work was small at baseline (*n* = 17) (Table 4, Supplementary Tables S3 and S4).

**Table 4:** Contextual factor analysis by group and time.

	*N* (%) at baseline	Difference between intervention and control (SE)	Adjusted difference in means (95% CI)	*P*-value for the interaction
Age 12 months	249	0.0 (0.3)	0.3 (−0.5; 1.1)	0.41
Age 36 months		−0.2 (0.4)	0.2 (−0.7; 1.1)	0.73
Gender 12 months:				
Male	45	−11.0 (6.4)	1.1 (−17.3; 19.6)	0.91
Female	202	−0.2 (3.2)		
Gender 36 months:				
Male		−11.5 (7.8)	0.4 (−20.1; 20.9)	0.97
Female		−1.4 (3.6)		
Disease duration 12 months:				
≤2 years	81	0.3 (5.0)	−4.2 (−19.6; 11.2)	0.59
˃2 years	168	−2.9 (3.4)		
Disease duration 36 months:				
≤2 years		2.0 (5.7)	−8.5 (−25.2; 8.3)	0.32
˃2 years		−5.4 (4.0)		
Comorbidities 12 months:				
0	56	0.3 (5.6)	1.6 (−15.3; 18.4)	0.86
≥1	193	−2.7 (3.3)		
Comorbidities 36 months:				
0		−6.0 (6.7)	8.6 (−9.9; 27.1)	0.36
≥1		−2.2 (3.7)		
SF12v2 Physical Component Score (0–100) 12 months	249	−0.2 (0.4)	−0.2 (−1.2; 0.7)	0.63
SF12v2 Physical Component Score (0–100) 36 months		−0.2 (0.4)	−0.2 (−1.3; 0.8)	0.67
SF12v2 Mental Component Score (0–100) 12 months	249	0.2 (0.3)	−0.1 (−0.9; 0.7)	0.85
SF12v2 Mental Component Score (0–100) 36 months		−0.1 (0.4)	−0.3 (−1.2; 0.6)	0.45
RAID-Total (0–10)12 months	249	−0.2 (1.5)	0.5 (−3.5; 4.5)	0.80
RAID-Total (0–10) 36 months		−1.2 (1.8)	−0.5 (−5.0; 4.0)	0.83
RADAI-5 (0–10) 12 months	249	−1.2 (1.6)	1.3 (−2.8; 5.4)	0.53
RADAI-5 (0–10) 36 months		−1.2 (2.0)	1.4 (−3.2; 6.0)	0.55
ONS-Manual Work 12 months:				
Non-manual	133	3.3 (3.9)		0.27
Manual	116	−7.5 (4.0)	−8.1 (−22.4; 6.2)	
ONS-Manual Work 36 months:				
Non-manual		−5.6 (4.3)		0.27
Manual		0.5 (4.9)	8.8 (−6.9; 24.4)	
RA-WIS (0–23) 12 months:				
Low	23	−12.5 (8.5)		
Moderate	119	−0.9 (4.1)	6.4 (−19.3; 32.1)	0.62
High	107	−0.1 (4.2)	−13.5 (−40.8; 13.9)	0.34
RA-WIS (0–23) 36 months:				
Low		2.2 (9.8)		
Moderate		−6.0 (4.5)	20.0 (−6.0; 46.0)	0.13
High		0.6 (5.2)	6.0 (−22.0; 33.9)	0.67
Work self-efficacy total (0–30) 12 months	249	0.8 (0.6)	0.3 (−1.2; 1.7)	0.70
Work self-efficacy total (0–30) 36 months		−0.3 (0.7)	0.8 (−2.4; 0.9)	0.40
Work demands: control over work schedule 12 months				
Somewhat—a great deal	128	2.1 (4.0)		
A little—not at all	121	−5.7 (4.0)	−10.2 (−24.6; 4.1)	0.16
Work demands: control over work schedule 36 months				
Somewhat—a great deal		−4.8 (4.3)		
A little—not at all		−0.9 (5.0)	1.5 (−14.3; 17.2)	0.85
Work demands: job flexibility 12 months				
Somewhat—a great deal	94	−3.0 (4.5)		
A little—not at all	155	−1.0 (3.6)	−4.8 (−19.4; 9.8)	0.52
Work demands: job flexibility 36 months				
Somewhat—a great deal		−4.1 (4.9)		
A little—not at all		−2.7 (4.4)	−5.4 (−21.1; 10.4)	0.50
Work demands: ability to postpone work tasks 12 months				
Somewhat—a great deal	64	5.7 (5.7)		
A little—not at all	185	−4.4 (3.3)	−9.5 (−26.0; 7.0)	0.26
Work demands: ability to postpone work tasks 36 months				
Somewhat—a great deal		−4.1 (6.2)		
A little—not at all		−3.5 (3.9)	1.8 (−15.7; 19.4)	0.84
Work demands receive help from colleagues 12 months				
Somewhat—a great deal	87	2.3 (4.9)		
A little—not at all	159	−4.4 (3.5)	−10.8 (−26.0; 4.4)	0.17
Work demands: receive help from colleagues 36 months				
Somewhat—a great deal		2.7 (5.4)		
A little—not at all		−7.9 (4.2)	−14.6 (−31.1; 1.8)	0.08
Work demands: physically demanding job 12 months				
Somewhat—a great deal	125	−2.0 (4.0)		
A little—not at all	124	−2.0 (4.1)	4.3 (−10.3; 18.8)	0.57
Work demands: physically demanding job 36 months				
Somewhat—a great deal		−2.7 (4.7)		
A little—not at all		−2.7 (4.6)	4.4 (−11.4; 20.2)	0.59
Work demands: mentally demanding job 12 months				
Somewhat—a great deal	232	−2.4 (2.9)		
A little—not at all	17	4.8 (10.2)	3.7 (−24.1; 31.6)	0.79
Work demands: mentally demanding job 36 months				
Somewhat—a great deal		−1.3 (3.4)		
A little—not at all		−30.9 (14.0)	40.4 (6.7; 75.0)	0.02

Notes: Disease duration ≤2 years = early disease, >2 years = established disease. Work Demands (5-point Likert scale, re-categorised as a little/not at all *vs* somewhat/quite a bit/a great deal). ONS: Office for National Statistics; RAID: Rheumatoid Arthritis Impact of Disease; RADAI-5: Rheumatoid Arthritis Disease Activity Index-5; RA-WIS; Rheumatoid Arthritis—Work Instability Scale; SF-12v2: Short Form 12v2 Health Survey; WALS: Work Activity Limitations Scale; WLQ-25PDmod: Work Limitations Questionnaire-25: Modified Physical Demands scale (i.e. items are not reverse scored); WPAI Work Productivity Activity Impairment.

### Cost-effectiveness analysis

There was little observed between-group difference in either costs or outcomes (Table 5). The mean INMB from 1000 bootstrapped resamples was −£196 (pseudo 95% CI: −£1712 to £1239). Over 40% of bootstrapped ICERs indicated that JRVR was more expensive and less effective (Supplementary Fig. S1). The probability that the intervention would be considered cost-effective under the current decision rules was 6.38% (Supplementary Fig. S2).

**Table 5:** Cost-effectiveness analysis results.

	Control	Intervention	Incremental (intervention–control)
Costs	*n* = 106	*n* = 98	
Intervention cost—mean (s.d.)	£0 (0)	£105.77 (55.57)	£105.77
Per person resource use—mean (s.d.)	£6565.62 (6781.73)	£6005.68 (6031.49)	−£559.94
Total per person costs (unadjusted)—mean (s.d.)	£6565.62 (6781.73)	£6113.69 (6034.47)	−£451.93
Total per person costs (adjusted for previous use)^a^	–	–	£346.24
QALYs	*n* = 103	*n* = 92	
Per person QALYs (unadjusted)	0.51	0.54	0.03
Per person QALYs (adjusted for baseline HRQoL)^a^	–	–	−0.003
Incremental Net Benefit (INB)			
Mean^b^ Incremental Net Benefit (pseudo 95% CI)^a^	–	–	−£195.86 (−£1711.54 to £1238.66)

a Using imputed data—*n* = 249.

b 1000 bootstrapped INB estimates.

HRQoL: health-related quality of life; INB: Incremental Net Benefit; QALY: Quality-Adjusted Life Year.

### Adverse events

No adverse events resulting from trial participation were reported.

## Discussion

This study found no evidence of a clinically important effect of the WORKWELL JRVR intervention on work participation outcomes at 6, 12 or 36 months. Differences between groups were small across all time points and outcomes, and consistent across sensitivity analyses. From an NHS and Social Care perspective, WORKWELL is unlikely to be cost-effective within this study, given the higher associated costs without improvement in HRQoL. The findings suggest that, within this cohort of individuals with InfA who had relatively short disease duration and were largely established in employment, the WORKWELL JRVR intervention did not provide additional benefit beyond usual care. The high proportion of participants remaining in work throughout the study and relatively stable employment characteristics at baseline may have limited the scope for improvement.

In this study, no meaningful differences were observed between groups across work outcomes, despite a structured and tailored intervention. The high employment retention and relatively stable baseline employment characteristics suggest limited scope for improvement in this population. Previous trials of JRVR have reported mixed findings, with some showing benefits in specific contexts. Participant interviews in the present study suggested that factors such as the timing of the intervention, workplace support, and flexibility of delivery may influence effectiveness. Participants with longer disease duration may already have adapted their work roles, reducing the potential additional impact of the intervention [48].

To further understand these findings, process evaluation data provide additional context. The intervention was well received by therapists and participants and showed good fidelity during delivery [48, 49]. Therapist interviews suggested that, although positive about remote delivery, additional workload pressures arising from the pandemic may have reduced its impact [49]. These insights highlight the importance of adaptable, co-designed, tailored solutions, for which further research is needed.

The high employment retention rate at 36 months in both groups (over 80%) indicates that most participants remained in work throughout the study, which may have limited the potential for additional benefit from the WORKWELL JRVR intervention. Both groups also received written self-help advice, which may have contributed to maintaining work participation. Process evaluation findings indicated that the self-help pack helped participants in understanding their employment rights and prompted some to seek additional work support [48].

Our study was large, pragmatic, focused on people with medium to high risk of work instability, and included an economic evaluation. However, the COVID-19 pandemic had a major impact throughout the trial. Many participants were WRFH, with potential benefits from reduced commuting, increased work flexibility, and ability to self-manage their condition, but also disadvantages of increased stress and poorer work facilities. Others were furloughed, and essential workers found their jobs increasingly pressured [50]. JRVR delivery was affected and JRVR completion rates reduced, potentially masking JRVR benefits.

There were some limitations. The WLQ-25PDmod, WALS and WPAI ask about recent (1–2 weeks) work difficulties. If participants were not working, furloughed or on long-term sick leave at follow-ups, these could not be completed, limiting the capturing of presenteeism changes. Many participants were white and on permanent contracts in large organizations, and the sample thus does not fully reflect the working population. A number of assumptions had to be made for costing NHS resource use due to an absence of data. This could have had an impact on the cost estimates for both interventions. There may also have been wider societal impacts and effectiveness that we could not measure in this RCT.

This trial demonstrated the feasibility of delivering tailored JRVR within routine rheumatology services, even during a pandemic. The findings, in the light of the participant interviews, raised questions about the optimal design and timing of JRVR, which may be more effective earlier in the disease course, or at key employment transition points. Furthermore, scalable approaches combining interactive digital tools, short interventions, and tailored self-guided resources could help meet diverse needs across clinical and occupational settings.

In conclusion, although WORKWELL JRVR did not significantly improve work outcomes, it represents an important step in testing pragmatic JRVR within the NHS. Future research should focus on personalization, digital delivery, and integration with employer-facing support to enhance sustainability and effectiveness.

Ethical approval was received from the Health Research Authority West Midlands—Solihull Research Ethics Committee 15 November 2018 (18/WM/0327) and the University of Salford Research, Enterprise, and Engagement Ethical Approval Panel (HSR1819-010). The trial was retrospectively registered: ClinicalTrials.gov NCT03942783 (registered 8 May 2019); ISRCTN Registry ISRCTN61762297 (registered 13 May 2019).

## Supplementary Material

keag373_Supplementary_Data

## Data Availability

The data underlying this article will be shared on reasonable request to the corresponding author.
