# Supplementary material for: The WORKWELL programme: a randomized controlled trial of job retention vocational rehabilitation for people with inflammatory arthritis
Source: Rheumatology (Oxford). 2026 Jul 17;65(8):keag373. doi: 10.1093/rheumatology/keag373 (PMC13451609; doi:10.1093/rheumatology/keag373)
Supplement: Supplementary file 1 [file keag373_supplementary_data.zip › rhe-25-2984-File002.docx]

**Supplementary Data S1: Lay Summary**

People with inflammatory arthritis, such as rheumatoid arthritis, often struggle at work due to pain, tiredness, and other symptoms. This can lead to people missing or stopping working.

The WORKWELL programme offers tailored, one-to-one support from occupational therapists. It was created to help people with inflammatory arthritis keep working. Together, they consider what makes work difficult and find ways to make it easier. These include job changes, learning to manage their condition, and knowing their work rights.

In this study, 249 people with inflammatory arthritis took part in a clinical trial across 18 NHS hospitals. Half received WORKWELL and a self-help pack. The other half received the self-help pack only. Researchers followed up both groups after 6, 12, and 36 months to see how they were doing at work.

The study found no big differences between the two groups, although at 12 months the WORKWELL group tended to take less time off sick. At 36 months, more were still working (93% versus 85% self-help group). The study was affected by the COVID-19 pandemic, which affected delivery and attendance.

This suggests more flexible ways to support people with arthritis at work, like online tools and tailored self-help options, could help.
