# Supplementary material for: The WORKWELL programme: a randomized controlled trial of job retention vocational rehabilitation for people with inflammatory arthritis
Source: Rheumatology (Oxford). 2026 Jul 17;65(8):keag373. doi: 10.1093/rheumatology/keag373 (PMC13451609; doi:10.1093/rheumatology/keag373)
Supplement: Supplementary file 1 [file keag373_supplementary_data.zip › rhe-25-2984-File003.pdf]

Supplementary Figure 1: Cost-effectiveness plane

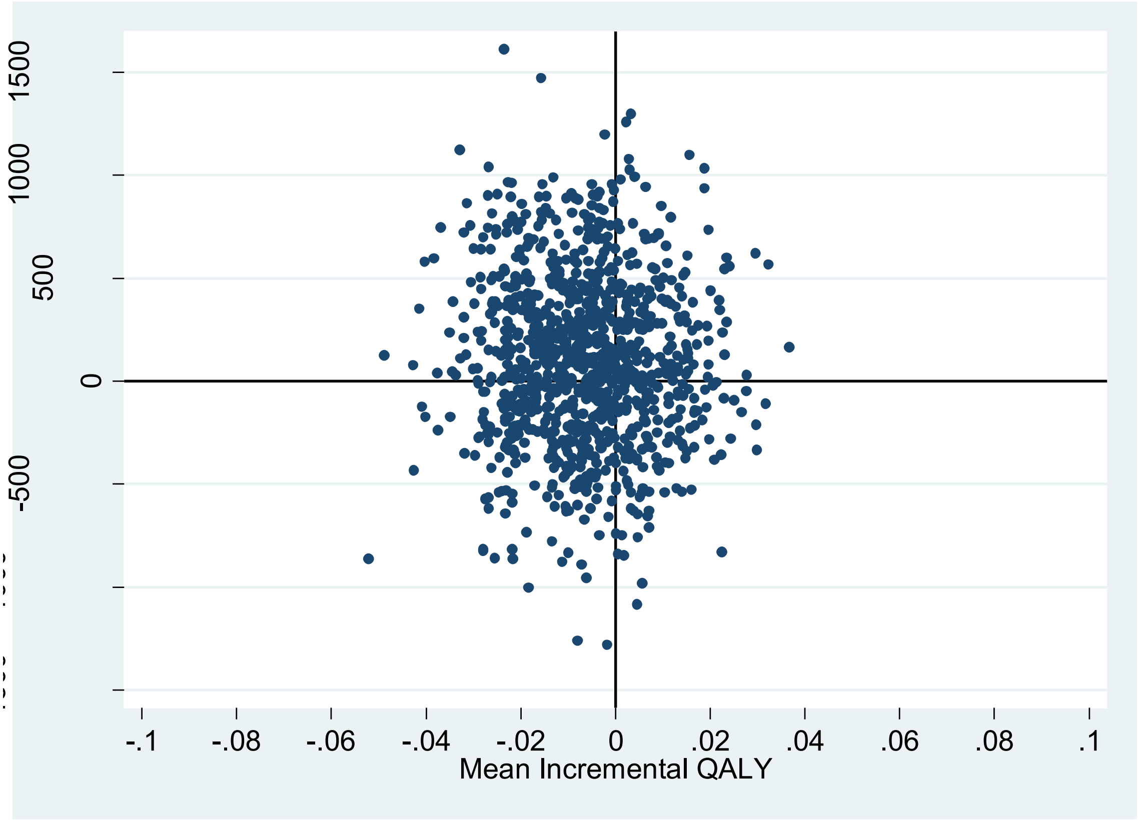

Supplementary Figure 2: Cost-effectiveness acceptability curve

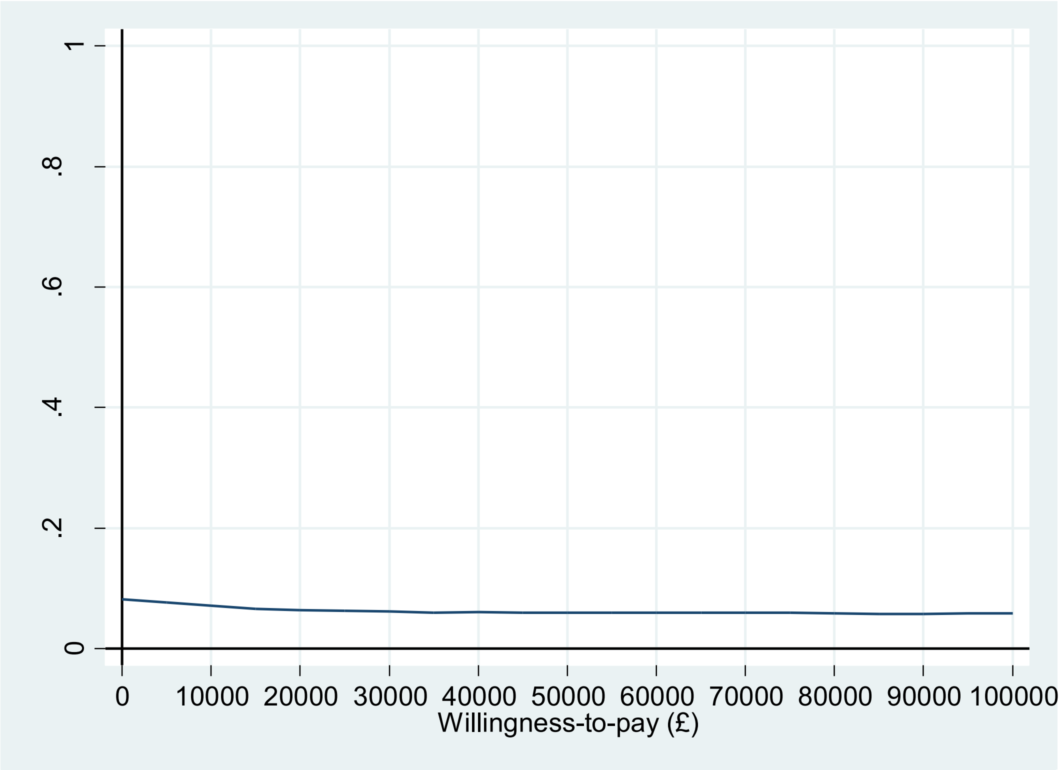

Supplementary Table 1: No. intervention participants treated: pre-trial pause; paused mid-treatment; post-trial restart (n = 124)

When Treated:	n	With-drew pre-treatment	Withdrew during treatment	DNA any treatment	DNA during treatment	Unable complete treatment by 12m	Completed except TR	Completed treatment including TR	Completed (+/- TR)	Withdrew post-treatment
**Pre-trial pause**	33 (27%)	0	1 (3%)	2 (6%)	0	n/a	1 (3%)	29 (88%)	30 (91%)	3 (9%)
**Paused mid-treatment**	36 (29%)	0	1 (3%)	0	3 (8%)	8 (22%) [a]	5 (14%)	19 (53%)	24 (67%) [c]	2 (5%)
**Post-trial restart**	55* (44%)	2 (4%)	2 (4%)	4 (7%)	4 (7%)	1 (2%) [b]	6 (11%)	36 (65%)	42 (76%)	1 (2%)
**TOTAL (n)**	124	2 (2%)	4 (3%)	6 (5%)	7 (6%)	9 (7%)	12 (9%)	84 (68%)	96 (77%)	6 (5%)

Key: % based on row total.

TR = telephone review**;** * = includes 12 delayed participants, referred pre-pause but not yet treated and subsequently re-referred when site re-opened.

[a] = unable to complete: 4 = long term sick leave; 4 = site not re-opened prior to participant’s 12m follow-up.

[b] = unable to complete: 1 = long term sick leave.

[c] = for 10/24, main treatment completed pre-pause and TR completed after re-start.

Supplementary Table 2: WLQ-25PDmod summed score at 12 months sensitivity analyses

N = 219	Adjusted difference in means	95% CI	p-value
Primary analysis	-1.8	-7.4 to 3.8	0.53
Sensitivity analyses			
i) per-protocol [1]	-2.7	-8.3 to 2.9	0.35
ii) per-protocol [2]	-3.3	-9.0 to 2.4	0.26

[1] Excludes those in the intervention group who did not receive any therapy (n=8) and those in the control group who stated that they had seen a therapist as part of the WorkWell trial (n=4).

[2] Excludes those in the intervention group who did not receive at least 2 sessions of therapy (n=15) and those in control group who stated that they had seen a therapist as part of the WorkWell trial (n=4).

Supplementary Table 3: Baseline characteristics for contextual factors

	Control	Intervention
Age, mean (SD)	48.3 (10.2)	48.8 (9.5)
Gender, N (%) Female	105 (84.7%)	97 (78.9%)
Disease duration, N (%) ≤2 years	45 (36.0%)	36 (29.0%)
Comorbidities, N (%) ≥1	94 (75.2%)	99 (79.8%)
RADAI-5 (0-10), mean (SD)	6.1 (1.8)	5.6 (1.8)
RAID-Total (0-10), mean (SD)	6.2 (1.7)	5.8 (1.8)
SF12v2 Physical Component Score (0 – 100) mean (SD)	36.5 (7.5)	36.9 (7.9)
SF12v2 Mental Component Score (0 – 100), mean (SD)	41.0 (9.1)	41.6 (9.7)
ONS- Manual Work, N (%)	58 (46.4%)	46.77 (46.8%)
RA-WIS, N (%):		
-Low	10 (8.0%)	13 (10.5%)
-Moderate	63 (50.4%)	56 (45.2%)
-High	52 (41.6%)	55 (44.4%)
Work Self-Efficacy Total, mean (SD)	17.9 (5.3)	18.0 (5.5)
Work Demands Control over Work Schedule, N (%) a little – not at all	61 (48.8%)	60 (48.4%)
Work Demands Job Flexibility, N (%) a little – not at all	73 (58.4%)	82 (66.1%)
Work Demands Ability to Postpone Work Tasks, N (%) a little – not at all	89 (71.2%)	96 (77.4%)
Work Demands Receive help from colleagues, N (%) a little – not at all	74 (60.7%)	85 (68.6)
Work Demands Physically demanding job, N (%) somewhat – a great deal	53 (42.4%)	72 (58.1%)
Work Demands Mentally demanding job, N (%) somewhat – a great deal	117 (93.6%)	115 (92.7%)

ONS: Office for National Statistics; RAID: Rheumatoid Arthritis Impact of Disease; RADAI-5: Rheumatoid Arthritis Disease Activity Index-5; RA-WIS; Rheumatoid Arthritis - Work Instability Scale; SF-12v2: Short Form 12v2 Health Survey; WLQ-25PDmod: Work Limitations Questionnaire-25: Modified Physical Demands scale (i.e. items are not reverse scored).

Supplementary Table 4: Contextual factors analysis: Mean (SD) WLQ25PDmod scores at baseline, 12 and 36 months by intervention group and variable category

	Baseline		12 months		36 months	
	**Control**	**Intervention**	**Control**	**Intervention**	**Control**	**Intervention**
Gender:						
Male	29.9 (22.2)	25.2 (18.7)	39.9 (31.1)	28.9 (22.4)	38.9 (34.4)	22.8 (10.4)
Female	31.0 (16.1)	31.9 (19.8)	31.2 (20.6)	30.8 (21.4)	34.5 (22.4)	33.4 (23.0)
Disease duration:						
≤2 years	30.1 (17.6)	31.5 (20.9)	28.6 (20.9)	30.6 (21.3)	30.9 (22.6)	33.5 (22.3)
>2 years	31.3 (16.8)	30.5 (19.5)	34.3 (23.3)	30.3 (21.8)	37.3 (25.4)	30.7 (21.8)
Comorbidities:						
0	27.1 (16.5)	26.4 (19.3)	26.2 (17.7)	26.5 (20.6)	28.8 (24.0)	24.3 (19.1)
≥1	32.1 (17.1)	31.9 (19.9)	34.7 (23.9)	31.6 (21.8)	37.3 (24.6)	33.8 (22.2)
ONS-Manual Work:						
Non-manual	32.5 (19.4)	32.8 (21.7)	29.5 (19.8)	33.9 (24.7)	35.9 (27.3)	30.1 (23.0)
Manual	29.0 (13.8)	28.5 (17.4)	35.6 (25.2)	26.5 (16.7)	34.4 (21.3)	33.8 (20.0)
RA-WIS, N (%):						
Low	14.0 (11.0)	12.4 (9.5)	31.3 (35.5)	18.9 (21.1)	11.0 (11.0)	17.0 (8.9)
Moderate	26.0 (13.0)	24.4 (14.0)	26.2 (17.1)	25.8 (21.4)	33.0 (25.8)	26.9 (19.8)
High						
Work Demands Control over Work Schedule:						
Somewhat – a great deal	30.4 (20.0)	31.6 (21.0)	28.7 (21.6)	32.5 (24.0)	32.7 (26.7)	28.9 (22.5)
A little – not at all	31.4 (13.5)	29.9 (18.7)	36.0 (23.2)	28.3 (28.3)	38.8 (21.2)	34.8 (20.8)
Work Demands Job Flexibility:						
Somewhat – a great deal	30.3 (17.7)	33.7 (20.9)	33.2 (22.5)	31.8 (23.6)	29.6 (20.4)	28.9 (20.3)
A little – not at all	31.3 (16.7)	29.3 (19.2)	31.8 (22.9)	32.9 (26.0)	37.37 (23.1)	33.5 (22.8)
Work Demands Ability to Postpone Work Tasks:						
Somewhat – a great deal	27.6 (15.9)	33.3 (22.9)	25.5 (12.8)	35.3 (28.2)	27.6 (23.8)	25.0 (20.7)
A little – not at all	32.2 (17.4)	30.0 (18.9)	35.1 (24.9)	29.1 (19.4)	39.0 (24.3)	33.5 (21.9)
Work Demands Receive help from colleagues,						
Somewhat – a great deal	27.8 (12.2)	31.2 (19.8)	28.7 (21.0)	34.7 (22.0)	28.3 (21.3)	33.2 (21.5)
A little – not at all	33.7 (19.1)	30.6 (20.0)	35.7 (23.3)	28.5 (21.2)	41.6 (25.1)	30.8 (22.1)
Work Demands Physically demanding job:						
A little – not at all	31.2 (17.7)	29.9 (18.1)	31.2 (22.8)	28.3 (22.8)	36.7 (26.6)	32.0 (22.3)
Somewhat – a great deal	30.4 (16.2)	31.4 (21.1)	33.9 (22.5)	31.9 (20.7)	33.3 (22.1)	31.2 (21.6)

	Baseline		12 months		36 months	
	Control	Intervention	Control	Intervention	Control	Intervention
Work Demands Mentally demanding job A little – not at all	31.7 (15.9)	23.6 (20.9)	27.0 (16.8)	26.2 (24.3)	49.0 (6.0)	12.9 (7.9)
Somewhat – a great deal	30.8 (17.2)	31.3 (19.7)	32.9 (23.0)	30.8 (21.4)	34.6 (24.9)	33.0 (21.9)

ONS: Office for National Statistics; RA-WIS; Rheumatoid Arthritis - Work Instability Scale.
